# Network-based identification of miRNAs and transcription factors and *in silico* drug screening targeting δ-secretase involved in Alzheimer's disease

**DOI:** 10.1016/j.heliyon.2021.e08502

**Published:** 2021-11-29

**Authors:** Saleem Iqbal, Md. Zubbair Malik, Debnath Pal

**Affiliations:** a Department of Computational and Data Sciences, Indian Institute of Science, Bangalore 560012, India; b School of Computational and Integrative Sciences, Jawaharlal Nehru University, New Delhi 110067, India

**Keywords:** Alzheimer's disease, miRNA, Systems medicine, δ-secretase, Molecular Dynamic Simulations, Biomarker

## Abstract

**Background:**

System medicine approaches have played a pivotal role in identifying novel disease networks especially in miRNA research. It is no wonder that miRNAs are implicated in multiple clinical conditions, allowing us to establish the hubs and nodes for network models of Alzheimer's Disease (AD). AD is an age-related, progressive, irreversible, and multifactorial neurodegenerative disorder characterized by cognitive and memory impairment and is the most common cause of dementia in older adults. Worldwide, around 50 million people have dementia, and there are nearly 10 million new cases every year. δ-secretase, also known as asparagine endopeptidase (AEP) or legumain (LGMN), is a lysosomal cysteine protease that cleaves peptide bonds C-terminally to asparagine residues in both amyloid precursor protein (APP) and tau, mediating the amyloid-β and tau pathology in AD. The patient's miRNA expression was found to be deregulated in the brain, extracellular fluid, blood plasma, and serum.

**Methods:**

Protein-Protein Interaction (PPI) networks of LGMN or δ-secretase were constructed using the Genemania database. Network Analyzer, a Cytoscape plugin, analyzed the network topological properties of LGMN. miRNAs related to Alzheimer's were extracted from the HMDD (Human microRNA Disease Database) and experimentally verified miRNA-gene interaction was obtained by searching miRWalk. Starbase v2.0 and miRanda were used for screening miRNA of LGMN genes. Moreover, to understand the regulatory mechanism in AD, we have screened major transcription factors of LGMN targeted genes using the Network Analyst 3.0, TRRUST (v2.0) server, and ENCODE. The Genotype-Tissue Expression (GTEx) and BEST tool were used to investigate the expression pattern of the LGMN gene. In parallel, we performed *in-silico* drug designing of the novel inhibitor scaffold of δ-secretase as powerful therapeutic targets by using the concept of scaffolds and frameworks. In this context, this study also aimed at identifying effective small molecule inhibitors targeting δ-secretase.

**Results:**

Among the 16 experimentally verified miRNAs, Network analysis of the LGMN and its associated miRNA identify novel hsa-miRNA-106a-5p and hsa-miRNA-34a-5p being more expressed in the brain. Our in silico high throughput screening, followed by XP docking revealed Oprea1 as the lead. Molecular dynamic simulations of the δ-secretase-docked complex have been carried out for a time period of 200 ns and revealed that Root Mean Square Deviation (RMSD) of the protein Cα-backbone with respect to its starting position increased to 1.20 Å for the first 25 ns of the trajectory and then became stable around 0.6 Å in the last 170 ns course of the simulation. The radius of gyration (RGYR) reveals that compactness was maintained till the end of simulations.

**Conclusion:**

Network analysis of LGMN associated miRNAs lead to the identification of two novel miRNAs, being highly expressed in the brain. This study also lead to the identification and expression of 10 Transcription factors associated with LGMN. Expression Heatmap results show high and continuous expression of LGMN in most of the regions of the brain, especially in the frontal cortex. Further, *in silico* drug analysis led us to the identification of Oprea1 which could be taken for further investigation to explore its potential for AD therapy.

## Highlights

• We have identified two novel miRNAs, viz., hsa-miRNA-106a-5p and hsa-miRNA-34a-5p being highly expressed in the brain.
• Topological analysis delineates LGMN as a hub (degree = 28), emphasizing that the network is dominated by LGMN.
• Major transcription factors of LGMN have been screened.
• Expression heatmap results show high and continuous expression of LGMN in most regions of brain, especially in frontal cortex.
• 200 ns molecular simulations of δ-secretase-Oprea1 docked complex revealed its high stability in terms of rmsd and Rgyr.

## Graphical abstract

**Figure:**
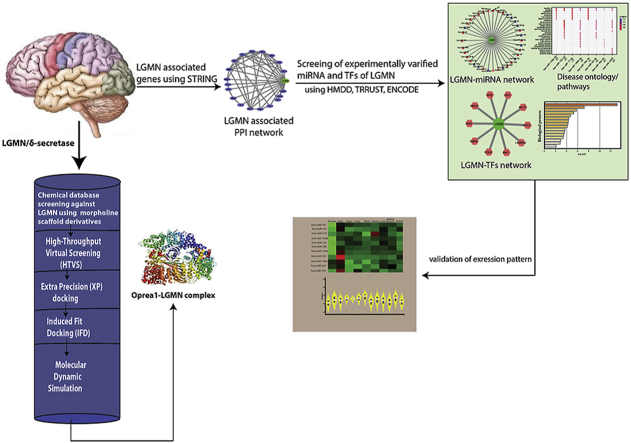

## 1 Introduction

Alzheimer's disease (AD) is the leading cause of dementia, is defined as a progressive neurodegenerative disease with neuropathological hallmarks: Neurofibrillary tangles and beta-amyloid plaques [1]. Worldwide, around 50 million people have dementia, and there are nearly 10 million new cases every year, and Alzheimer's disease is the most common form of dementia and may contribute to 60–70% of cases (https://www.who.int/news-room/fact-sheets/detail/dementia). In the USA alone, it is estimated that by mid-century, the population aged 65 and older with Alzheimer's dementia may grow to 13.8 million, representing a steep rise in estimates compared to 2020 [2]. The neurofibrillary tangles formed by hyperphosphorylated Tau protein lose their physiological role in promoting microtubule stability in neurons [3, 4]. The phenotypic spectrum in AD patients includes memory loss as well as a decline in other cognitive domains (e.g., executive function, language, perceptual-motor), functional decline, and especially in later stages neuropsychiatric symptoms (e.g., irritability, depression, agitation, and hallucinations) [5]. The secretases viz., α-Secretase, β-secretase, and γ-secretase are proteases that control the production of amyloid-β (Aβ) in the brain and represent the most promising drug targets for Alzheimer disease therapies.

Among Secretases, a new class of secretase dubbed as δ-secretase has been in focus as a therapeutic target for AD recently. It has been found that δ-secretase is progressively upregulated and activated during aging in the mouse brain. Furthermore, human AD brains show high elevation and activation of δ-secretase, compared to normal controls. Active δ-secretase cleaves both amyloid precursor protein (APP) and tau which play a major pivotal role in AD pathogenesis. Processing of APP by δ-secretase facilitates β-Site amyloid precursor protein cleaving enzyme 1 (BACE1) to cleave APP, leading to Aβ upregulation [6]. Moreover, the Aβ hypothesis and the tau hypothesis are the two commonly accepted hypotheses based on APP and Tau pathological characteristics. The amyloid cascade hypothesis suggests that the imbalance between the production and clearance of Aβ is the key trigger of a cascade of events that leads to AD [7, 8]. As AD remains incurable at present despite decades of research efforts, therapeutic intervention with disease-modifying agents to reverse or slow down the neurodegeneration has drawn much attention [9].

### 1.1 MicroRNAs and AD

MicroRNAs are involved in many biological processes and diseases, particularly multifactorial diseases, providing an excellent tool to probe their mechanisms [10]. MicroRNAs, a class of non-coding RNAs, have been acknowledged as important regulators for post-transcriptional gene expression by either repressing translation or degrading target mRNAs [11]. Post the discovery of microRNAs, they have been identified as the regulators most frequently implicated in many critical biological events, such as development, growth, differentiation, and neurodegenerative processes [12, 13]. Numerous microRNAs (miRNAs) have been considered as key players in the regulation of neuronal processes, e.g. studies as explored by Quang et al., 2018, ameliorate that miR-25 may suppress Kruppel-like factor 2 (KLF2) and stimulate the nuclear factor- E2-related factor 2 (Nrf2) pathway, which further aggravates hippocampal neuron injuries induced by Aβ1-42 in mice with AD [14]. Studies have also demonstrated that the expression of several miRNAs changes in AD [15, 16, 17, 18]. The therapeutic potential of miR-155 via regulation of T cells in AD has been described [19]. Several miRNAs are expressed in the brain are involved in inflammation and microglia activation [20, 21, 22]. MicroRNAs play vital roles in neuronal development, synaptic plasticity, and neurodegeneration [23].

### 1.2 δ-Secretases and AD

δ-secretase, AEP, or legumain, hereafter denoted by LGMN in this study, is a lysosomal cysteine protease that cleaves peptide bonds C-terminally to asparagine residues in both APP and tau, mediating the Aβ and tau pathology in AD. It is a cysteine protease encoded by gene legumain represented by ‘LGMN’ and involved in various cellular events, including antigen processing, the cleavage of other lysosomal enzymes, osteoclast formation, and normal kidney function [24, 25, 26, 27, 28]. Biochemically, secretase is highly regulated by its specificity for asparagine residues and pH. Dysregulation of δ-secretase activity has been implicated in various diseases, including cancers and neurodegenerative diseases [29, 30, 31, 32, 33, 34].

We have investigated and analyzed the interactions of the LGMN target gene in the context of a network, as well as the other important genes providing a modular framework to it. The proposed analysis is focused on approaches to network analysis to predict several unknown AD-associated genes, which can be validated as reliable candidates through *in vitro*/*in vivo* experiments.

## 2 Materials and methods

### 2.1 PPI network construction of LGMN associated genes

#### 2.1.1 Construction of a protein-protein interaction (PPI) network

The network was constructed using the Genemania database [35] and the result file was verified and uploaded for further literature verification in Cytoscape [36]. The first analysis focused on the topological properties of the network to understand its structure, and the possible existence of hidden mechanisms. We calculated the degree distribution (P(k)), clustering coefficient (c(k)), neighborhood connectivity C_N_(k) and centrality betweenness (C_B_), closeness (Cc) using Cytoscape plugins, Network Analyzer [37], for LGMN and the associated protein interaction network. The degree is denoted by the means number of genes interacting with LGMN.

#### 2.1.2 Identification of miRNA associated with LGMN networked genes

Screening of miRNA of LGMN genes was performed using Starbase v2.0 [38], miRWalk v3.0 [39], and miRanda [40]. In this study, miRNAs related to Alzheimer's were extracted from the Human microRNA Disease Database (HMDD) and experimentally verified miRNA-gene interaction was obtained by searching miRWalk v3.0.

#### 2.1.3 Identification of transcription factors associated with the networked genes

Transcription Factors (TFs) are key trans-acting factors in transcriptional regulation. Therefore, explicating TF-target interactions is an important step to understand the regulatory mechanism in human diseases. In our study, we have screened major transcription factors of LGMN targeted genes using the Network Analyst 3.0 [41] and TRRUST (v2.0) server [42], ENCODE [43].

#### 2.1.4 Validation of the expression pattern

We used the GTEx portal [44] to obtain the expression of LGMN in different areas of the brain in form of a violin plot and organized the data based on the median. Also, a spatiotemporal expression heatmap is generated based on the calculated expression levels of LGMN in RNAseq data from Brainspan [45], through BEST, a web server for brain expression spatiotemporal pattern analysis [46]. We checked the expression data from the public database BrainEXP [47] and found that most of the contributing datasets did not collect agonal information.

*In silico* screening is efficient, and represents a simpler and less expensive method, as proven by several docking studies [48, 49]. Molecular frameworks introduced by Bemis and Murcko [50], attempt to organize the chemical known space to better predict the pharmacodynamic activity of a certain type of structure [50]. Moreover, the concept of scaffolds and frameworks is applied in drug discovery for the identification of classes of compounds, similarity searches, and many different virtual screening techniques [50].

#### 2.1.5 Repossession of LGMN inhibitors through shape-based screening

The compound 4-morpholin-4-yl-2,1,3-benzoxadiazol-7-amine (shortly referred to as ‘morpholine derivative’ in this manuscript), was used as an input structure for shape-based screening as the given compound was found to have a therapeutic effect of an orally bioactive and brain permeable δ-secretase inhibitor in mouse models of AD [51]. Briefly, the SMILE format and chemical structure of the given compound was retrieved from Pubchem, Swiss Similarity, CHEMBL, Natural Compound Database, online platforms which allows us to perform similarity search chemical hits with respect to our reference structure [52, 53, 54]. Structures of 1,86,607 compounds were retrieved and processed for high throughput screening, followed by XP docking, and Molecular Dynamics simulation analysis against the LGMN.

#### 2.1.6 Preparation of ligand and protein

Protein and ligand preparation were carried out as defined in (Iqbal et al., 2017) [55]. Briefly, Crystal Structure of δ-secretase (PDB ID: 5LU9) [52] was downloaded from the Protein Data Bank. Protein preparation was done by the wizard of Schrodinger 14-2 [56] where missing hydrogen and bond order was assigned followed by the refinement. Structural waters being vital in mediating hydrogen bonds between receptor and the ligand were retained at the center of the grid within 20Å edges of the catalytic site. All the compounds retrieved from various databases were prepared and energy minimized using the Ligprep module of the Schrodinger with probable tautomeric and ionization states at pH = 7 ± 1 followed by minimization with OPLS 2005 force field [57].

#### 2.1.7 Screening and Induced Fit Docking

Glide virtual screening within virtual screening workflow (VSW) module of Schrödinger and Glide molecular docking with extra precision level resulted only in screening compounds with good binding towards target proteins. The virtual screening and docking of 1,86,607 compounds from the Pubchem, CHEMBL, and Super Natural Database library of compounds was carried out using Schrödinger [58]. Molecular docking is a computational simulation that predicts the preferred orientation of a ligand with a receptor during their interaction to form a complex with higher stability. In this study, GLIDE was used to perform flexible, rigid, and Induced fit docking at the active site of δ-secretase for predicting the binding affinity and ligand efficiency to the target [58, 59].

#### 2.1.8 Molecular dynamics simulations

Molecular Dynamics simulations were carried out using the Desmond Software [60]. Briefly, Optimized Potentials for the Liquid Simulations (OPLS)-2005 force field were used in this system to determine the protein (δ-secretase) interactions with efficient ligand molecules and solvated with the simple point charged (TIP4P) water model. The orthorhombic water box was used to create a 10Å buffer region between the protein atoms and box sides. Systems were neutralized with Na^+^ ions. For energy calculation, the OPLS-2005 force field was used. The Martyna-Tobias-Klein scheme was used for pressure coupling. PME algorithm [61, 62] was used for calculating the electrostatic forces; all runs have been performed at 300K at constant volume and temperature (NPT ensemble) under certain periodic boundary conditions. The MD simulations analysis of δ-secretase lead complex were carried out for a period of 200 ns having 52,080 trajectories, which were recorded for every 2.0 ps. Root Mean Square Deviation (RMSD), Root Mean Square Fluctuation (RMSF), and potential energies were evaluated in this study.

#### 2.1.9 Normal mode analysis

We analysed our protein-ligand complexes and measured the parameters in terms of eigen value that determine relationship between protein structure and ligand complexes of Oprea1, Cocrystal and +ve control (Aloxistatin) via Normal Mode analysis (NMA) method using iMODS server [63]. The output of normal mode analysis is a collection of points corresponding to the location of atoms and associated motion vectors, where a vector at each point is known [63].

## 3 Results and discussion

### 3.1 PPI network construction of LGMN associated genes

**Figure 1:**
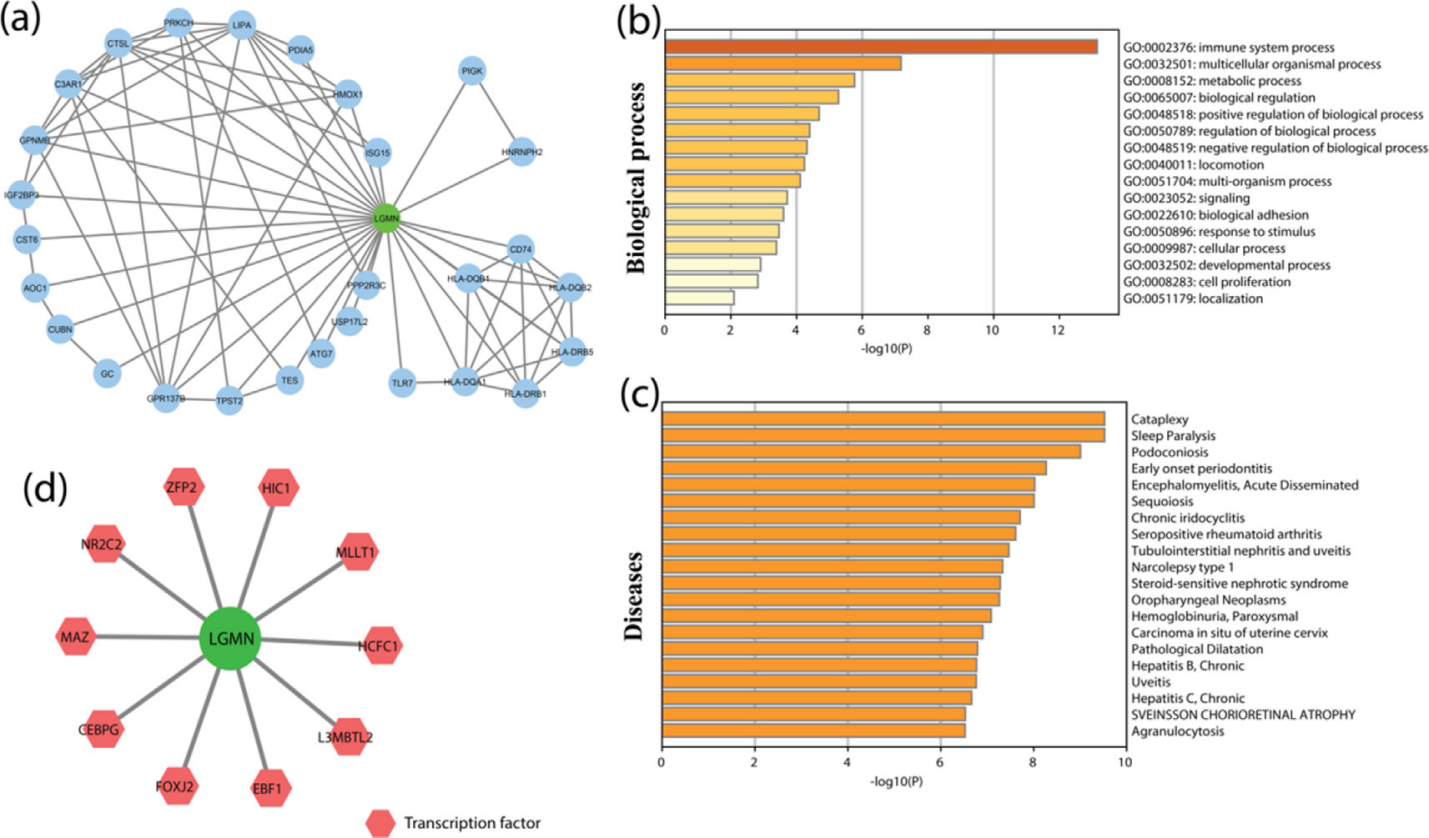
PPI Network construction (a) PPI network of LGMN and its associated protein. (b) Bar graph of the top-level Gene Ontology especially depicting the prevalence of Immune system biological processes of genes involved in LGMN regulatory network, colored by p-values. (c) Bar graph of disease enrichment of genes involved in LGMN regulatory network, colored by p-values (d) The hexagonal-shaped red nodes represent transcription factors (TFs) associated with LGMN.

The degree of LGMN in the network was found to be 28; i.e., 28 proteins interact with LGMN. The PPI network of LGMN and its associated proteins is shown in Figure 1a. For the given network, the LGMN with the highest degree node was identified as a hub. Therefore, the network is dominated by LGMN, so the structure, functioning, and control of the network are mainly performed by LGMN.

Hub genes in the network are the genes that are highly connected with other genes in the network on a direct basis. The hub gene (LGMN) plays an important role in maintaining and regulating the stability of the Alzheimer's disease network. The topological properties in terms of Centrality, Clustering Coefficient, Neighborhood Connectivity, Topological Coefficient, and Network density of LGMN associated PPI network are shown in Table 1. We conducted GO enrichment analysis to expound the potential biological process of genes involved in the LGMN regulatory network by using Metascape online server (https://metascape.org/gp/). LGMN and its associated genes were enriched in the biological process including localization, cell proliferation, developmental process, cellular process, response to stimulus, biological adhesion, signaling, multi-organism process, locomotion, negative regulation of the biological process, regulation of the biological process, positive regulation of the biological process, biological regulation, metabolic process and multicellular organismal process (Figure 1b) (Table S1). We observed that LGMN and its associated genes were involved in cataplexy, chronic iridocyclitis, sequoiosis, encephalomyelitis, acute, disseminated, early-onset periodontitis, podoconiosis, sleep paralysis (Figure 1c) (Table S1).

**Table 1:** Topological properties of LGMN.

Degree	28
Betweenness Centrality	0.793827
Closeness Centrality	1
Clustering Coefficient	0.126984
Neighborhood Connectivity	4.428571
Topological Coefficient	0.167582
Network Density	0.187

### 3.2 Identification of transcription factors associated with key genes

10 TFs were associated with LGMN protein; viz., L3MBTL2, FOXJ2, HIC1, EBF1, ZFP2, MAZ, NR2C2, CEBPG, MLLT1, and HCFC1, has been shown in (Figure 1d). The LGMN and associated TFs network were constructed and visualized using Cytoscape. These data support the finding that these genes may be important factors in AD, but there is a need for future analysis.

### 3.3 Validation of the expression pattern of the LGMN gene

**Figure 2:**
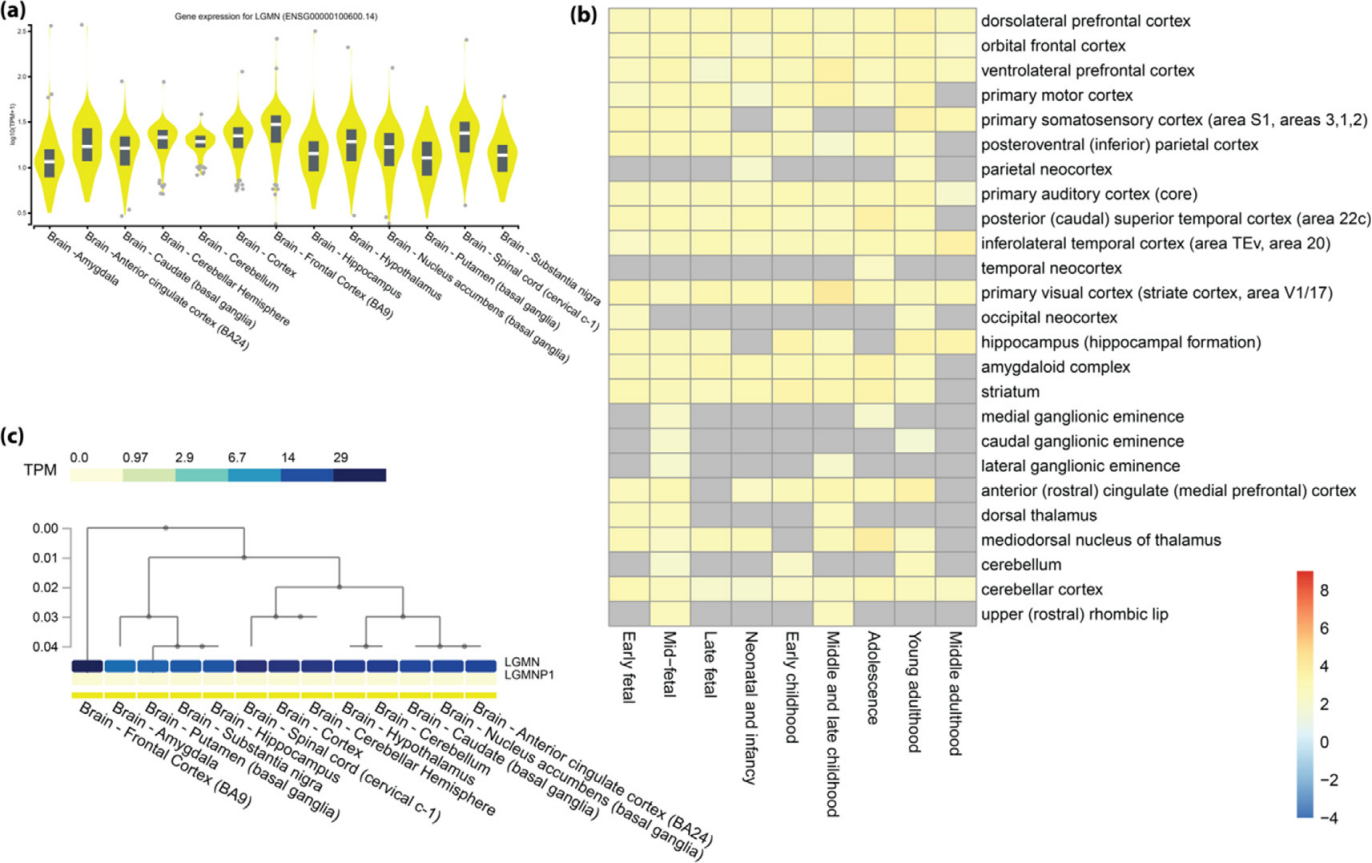
Expression level of LGMN (a) Violin plot from GTEx portal expression levels in different regions of the brain. (b) Spatio-temporal expression heatmap from BEST tool (c) heatmap shows the expression of LGMN in the brain.

To study the tissue-specific gene expression and regulation of LGMN in different areas of the brain (Figure 2a), we used the Genotype-Tissue Expression (GTEx) tool. The expression pattern of LGMN is analyzed and violin plots showing the expression distribution of LGMN in the brain are shown in Figure 2a. To investigate the expression pattern of the LGMN gene in different areas of the brain against the different age groups, BEST tool was used to study the Spatio-temporal expression heatmap. From the Spatio-temporal expression heatmap, it is evident that regions of the brain viz., dorsolateral prefrontal cortex, orbital frontal cortex, ventrolateral prefrontal cortex, inferolateral temporal cortex, primary visual cortex, and cerebellar cortex show significant expression of LGMN and can be depicted in Figure 2b. Both the results show high and continuous expression of LGMN in most of the regions of the brain, especially the frontal cortex. In the violin plot as represented in Figure 2a, the TPM (transcripts per million) is the highest, and in Figure 2b, we can see that LGMN is expressed at a different stage of the life cycle in the different portion of the brain; i.e., the cerebellum, frontal, parietal, temporal, and occipital cortexes are shown in Figure 2c. The LGMN is expressed throughout the life cycle in the cortex of dorsolateral prefrontal, orbital frontal, and ventrolateral prefrontal cortex.

### 3.4 Identification of miRNA associated with key genes

**Figure 3:**
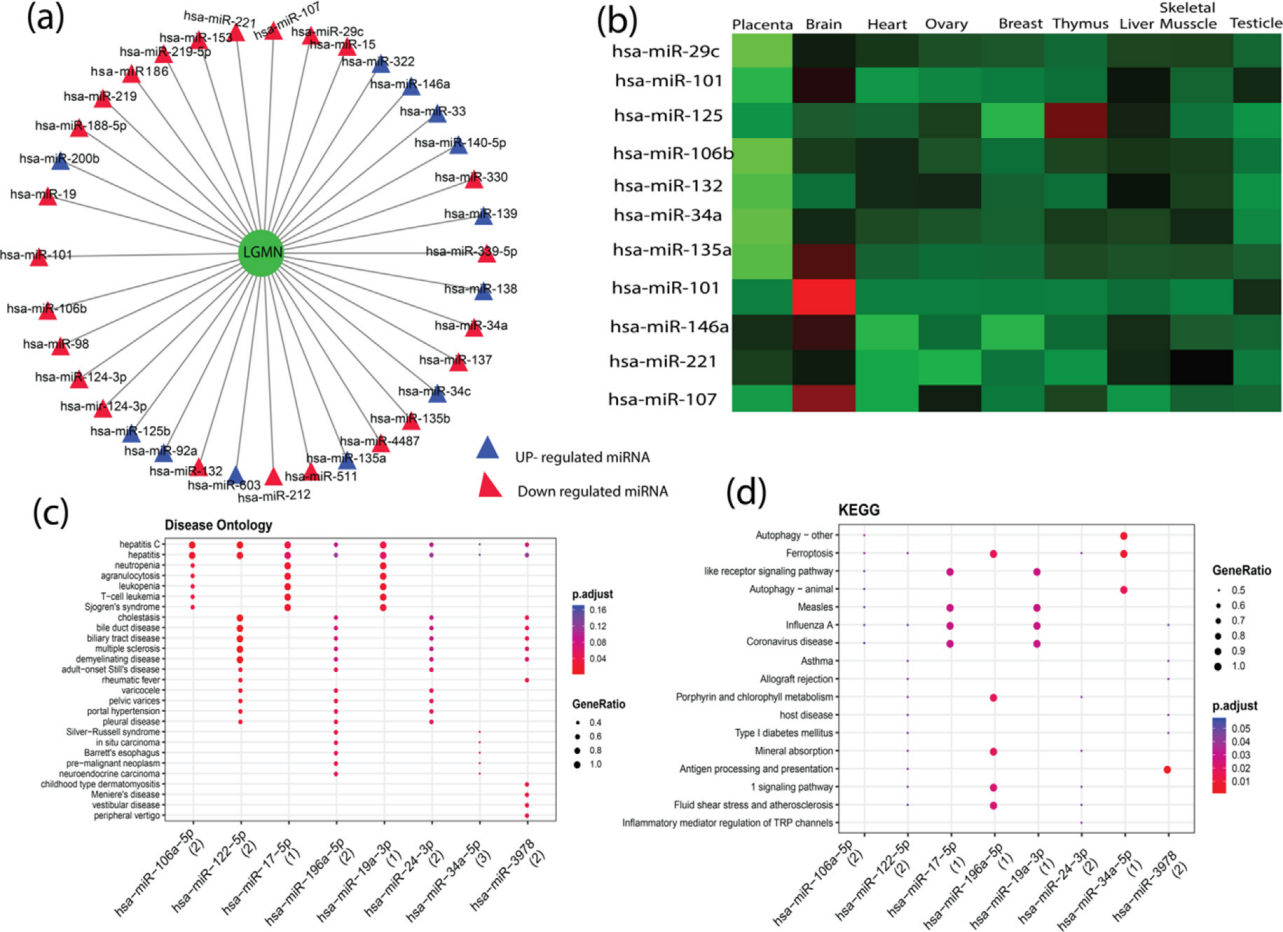
(a)The miRNA-gene regulatory network of AD-specific genes identified in brain datasets. The triangular-shaped cyan color node represents miRNAs (b) heatmap shows the expression of miRNA in different tissue (c) The most significantly biological ontology of miRNAs targeted to LGMN (d) biological pathway of the miRNAs targeted to LGMN.

The LGMN genes were associated with 16 experimentally validated miRNAs (Figure 3a) such as hsa-mir-26b-5p, hsa-mir-192-5p, hsa-let-7e-5p, hsa-let-7f-5p, hsa-mir-124-3p, hsa-mir-20a-3p, hsa-mir-217, hsa-mir-433-3p,hsa-mir-1-3p, hsa-mir-128-3p, hsa-mir-129-2-3p, hsa-mir-146a-5p, hsa-mir-194-5p, hsa-mir-23b-3p, hsa-mir-34a-5p, hsa-mir-375, etc (Figure 3a). The LGMN and associated miRNA network was constructed and visualized using Cytoscape. The miRNAs recognized by these databases were considered as candidate miRNAs and intersected with the LGMN. The miRNA expression pattern is shown in Figure 3b (Table S3) in the brain. Disease ontology of significant miRNA was performed using the MIENTURNET tools [64]. (MicroRNA ENrichment TURned NETwork) makes use of the holistic approach of the network theory to infer possible pieces of evidence (computational or experimental) of miRNA regulation by capturing topological properties of the miRNA-target regulatory network that would be not evident through a pairwise analysis of the individual components. The statistically most enriched GO terms were visualized in ggplot2 [65]. We observed that hsa-miRNA-106a-5p and hsa-miRNA-34a-5p are more expressed in the brain (Figure 3b) (Table S3).

In APP/PS1 mice, the level of has-miRNA-34a increased in tandem with the increase in amyloid (A); however, in miRNA-34a knockout mice, behavioral dysfunction was substantially reduced, owing to the inhibition of γ-secretase activity [66]. γ-secretase-mediated proteolytic cleavage produces NICD1, the active form of Notch1. As a consequence, blocking either this cleavage pathway or Notch1 causes NICD1 levels to drop [67]. The findings suggest that miR-34a affects the Notch signaling pathway [68]. The increase in miR-34a expression is linked to a decrease in Notch signaling. The hsa-miRNA-106a-5p showed the significant disease ontology terms related to T−cell leukemia, agranulocytosis, neutropenia, hepatitis, Sjogren's syndrome as depicted in (Figure 3c & 3d) (Table S4). The hsa-miR-106a-5p is a well-known miRNA that is highly investigated in uterine disease, breast carcinoma, and coronary artery disease. Notably, concerning AD susceptibility, hsa-miR-106a-5 and hsa-miR-106b-5p - miRNAs are found "upstream" of the genes linked to Alzheimer's disease. Increased Aβ secretion was observed when cellular cholesterol efflux was impaired by hsa-miR-106 suppression of ATP-binding cassette transporter A1 (ABCA1) [69]. Studies have also found that miR-106-5p inhibits Aβ42-induced tau phosphorylation at Tyr18 by targeting Fyn-tyrosine kinase [70, 71]. We have crosschecked the identified miRNA's with previous studies as carried out by Leidinger and coworkers in 2017 leading to the identification of blood-based miRNA viz., hsa-mir-26b-5p [72]. Surprisingly, as inferred from our analysis, we identified the same miRNA: ‘hsa-mir-26b-5p’ being dysregulated in the brain too and needs further investigation to decipher its role.

### 3.5 Molecular docking simulations

We explored the δ-secretase inhibitory bioactive molecules through molecular docking. Moreover, the concept of scaffolds and frameworks is applied in drug discovery for the identification of classes of compounds, similarity searches, and many different virtual screening techniques. 4-morpholin-4-yl-2,1,3-benzoxadiazol-7-amine was used as an input structure for shape-based screening as the given compound was found to have decreased the N368 truncation and phosphorylation of tau in tau P301S mice by inhibiting the cleavage of tau by δ-secretase, which led to the identification of Oprea1. The δ-secretase structure has a sequence length of 262 amino acids with a resolution of 2.27Å. We used structure similarity search of ‘morpholine derivative’ as an input compound against the δ-secretase. Glide energy which is an empirical scoring function that approximates the ligand binding free energy used to rank poses of different ligands, more negative values represent tight binders. The screening of bioactive compounds obtained from databases as mentioned above lead us to the identification of 20 compounds, all compounds were filtered based on glide energy. Out of which 3 compounds were put for XP docking, followed by identification of one lead after performing Induced Fit Docking (IFD) using glide energy as a filter. Corresponding to the lowest free energy (or highest score) provided by the Glide program the docked conformation was selected as the most probable binding pose of top compounds. In the current study, the identified bioactive compound IUPAC name: 3-(1,3-Dioxo-1,3,3a,4,7,7a-hexahydro-2H-4,7-methanoisoindol-2-yl)-N-{5-[(4-nitrophenyl)sulfonyl]-1,3-thiazol-2-yl}propenamide also dubbed as Oprea1 in the current study, exhibited a docking score of -13.64 and Glide Energy of -76.28 kcal/mol as shown in Table 2. Aloxistatin being an cysteine protease inhibitor was used as a +ve control in studying protein ligand interactions. The binding affinities were better than that of the input 4-morpholin-4-yl-2,1,3-benzoxadiazol-7-amine and +ve control (Aloxistatin) which bound with an affinity of -52.41 kcal/mol and -50.13 kcal/mol respectively.

**Table 2:** Induced fit docking results of the δ-secretase-Oprea1 complex.

Compound	Hydrogen Bonding Interactions	Hydrophobic Interactions	Docking Score	Glide energy (kcal/mol)
Oprea1	Thr 250, Hie 252, Hie 256, Lys 259, Gln 269	Met 268, Thr 274, Ser 276, Thr 277	-13.64	-76.28
Cocrystal	Gln 269, Thr 277	His 252, Lys 273, Thr 274, Ser 276	-7.26	-52.41
+ve Control (Aloxistatin)	Gln 269, His 256, Lys 259, Lys 273	His 252, Tyr 255, Met 268, Thr 277	-6.51	-50.13

**Figure 4:**
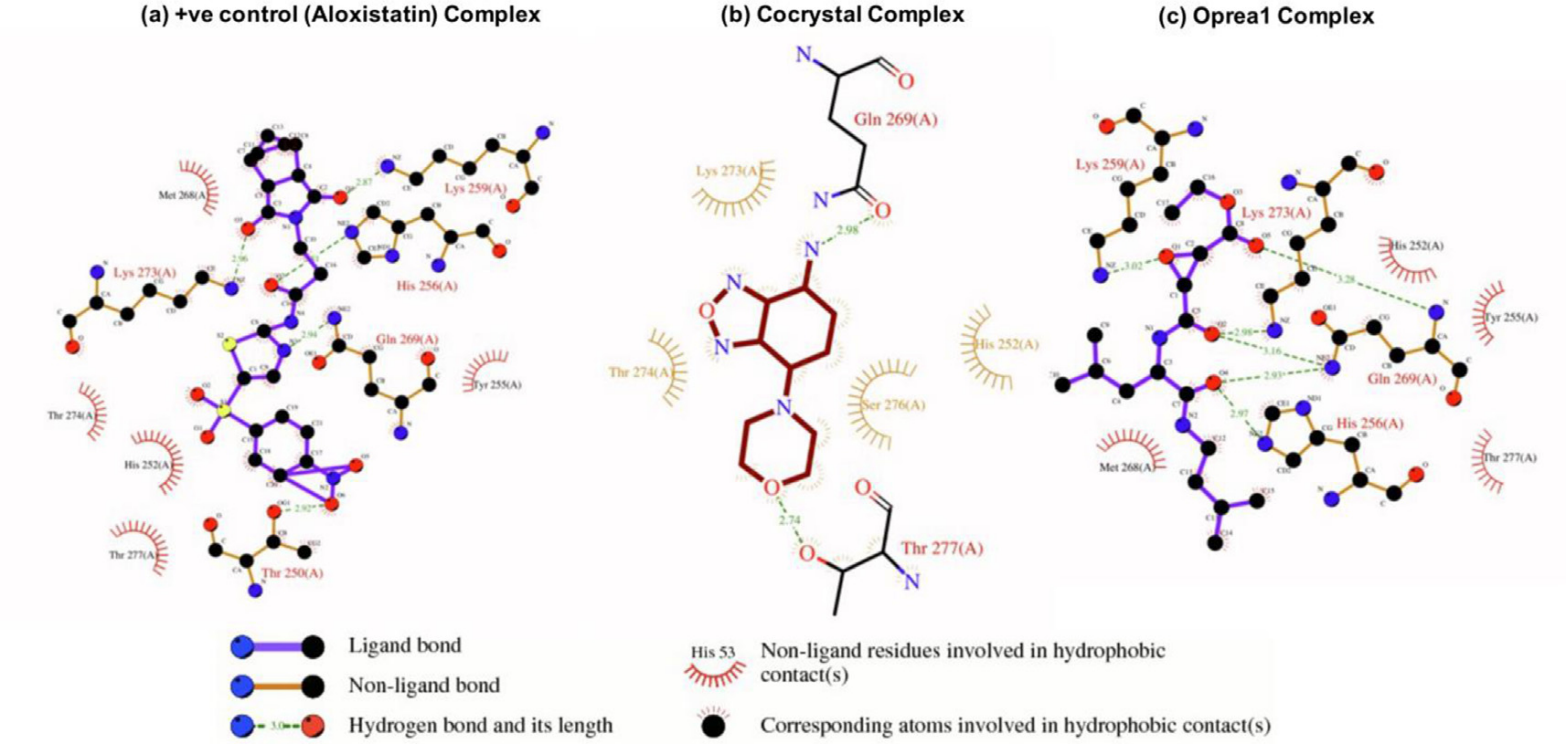
(a)-(c): 2D -Interaction diagram of the δ-secretase-Aloxistatin, Cocrystal & Oprea1 docked complex.

**Figure S1:**
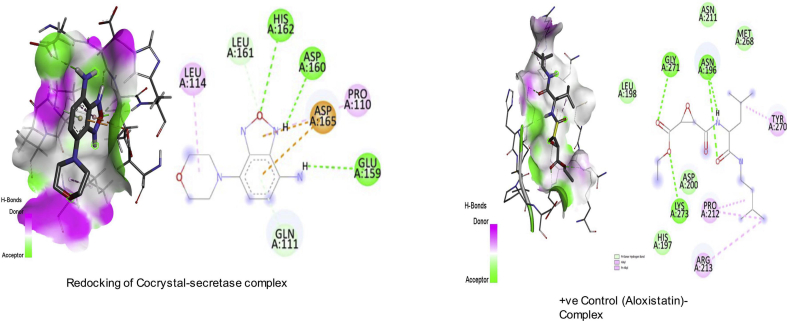
Interaction of Aloxistatin & cocrystal.

Hydrogen and hydrophobic interactions were analyzed using, Maestro Visualiser and PyMol (PyMOL Molecular Graphics System, Version 2.0 Schrödinger, LLC). Interactions of the δ-secretase-Oprea1 docked complex are shown in Table 2. IFD studies reveal that the Oprea1 bound well at the active site target. Figure 4a–c represent the interaction diagram of the δ-secretase-Aloxistatin, cocrystal-complex and Oprea1 docked complex respectively using LigPlot+ [73], wherein residues like Thr 250, Hie 256, Lys 259, Gln 269, Lys 273, Thr 277 as hydrogen-bonded while residues such as His 252, Tyr 255, Met 268, Lys 273, Thr 274, Ser 276, Thr 277 interacted hydrophobically. 3D active site interaction of +ve control (Aloxistatin) and cocrystal have been shown in Supplementary section (Figure S1). Based on the binding conformation and in orientation, Oprea1 bound in the same orientation as that of 4-morpholin-4-yl-2,1,3-benzoxadiazol-7-amine.

### 3.6 Molecular dynamics simulations

**Figure 5:**
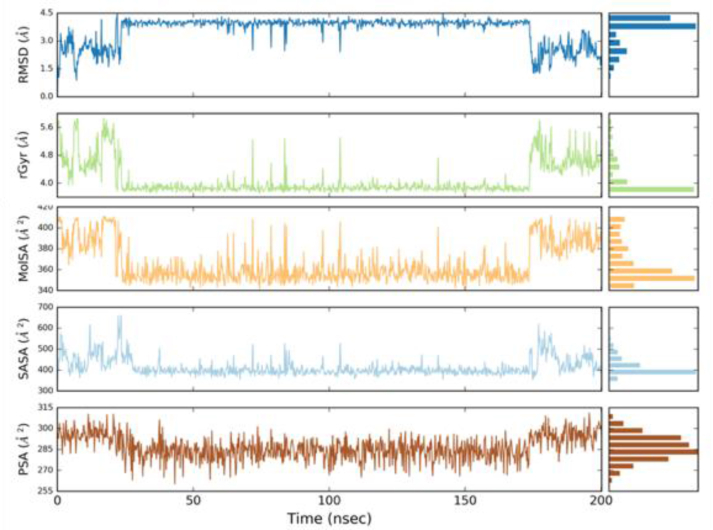
Ligand properties of Oprea1 in terms of PSA, SASA, and rGYR in terms of PSA, SASA, and rGYR into binding pocket δ-secretase.

To study the steady nature and conformational stability of δ-secretase, molecular dynamics simulations (MDS) of the δ-secretase-docked complex have been carried out for a time period of 200 ns. The trajectories were visualized and analyzed based on the trajectories of the reformed simulations. Ligand properties in terms of Polar Surface Area (PSA), Solvent Accessible Surface Area (SASA), and Radius of Gyration (rGYR) have been shown in Figure 5, illustrating the stability of the δ-secretase-docked complex into the δ-secretase binding pocket.

**Figure 6:**
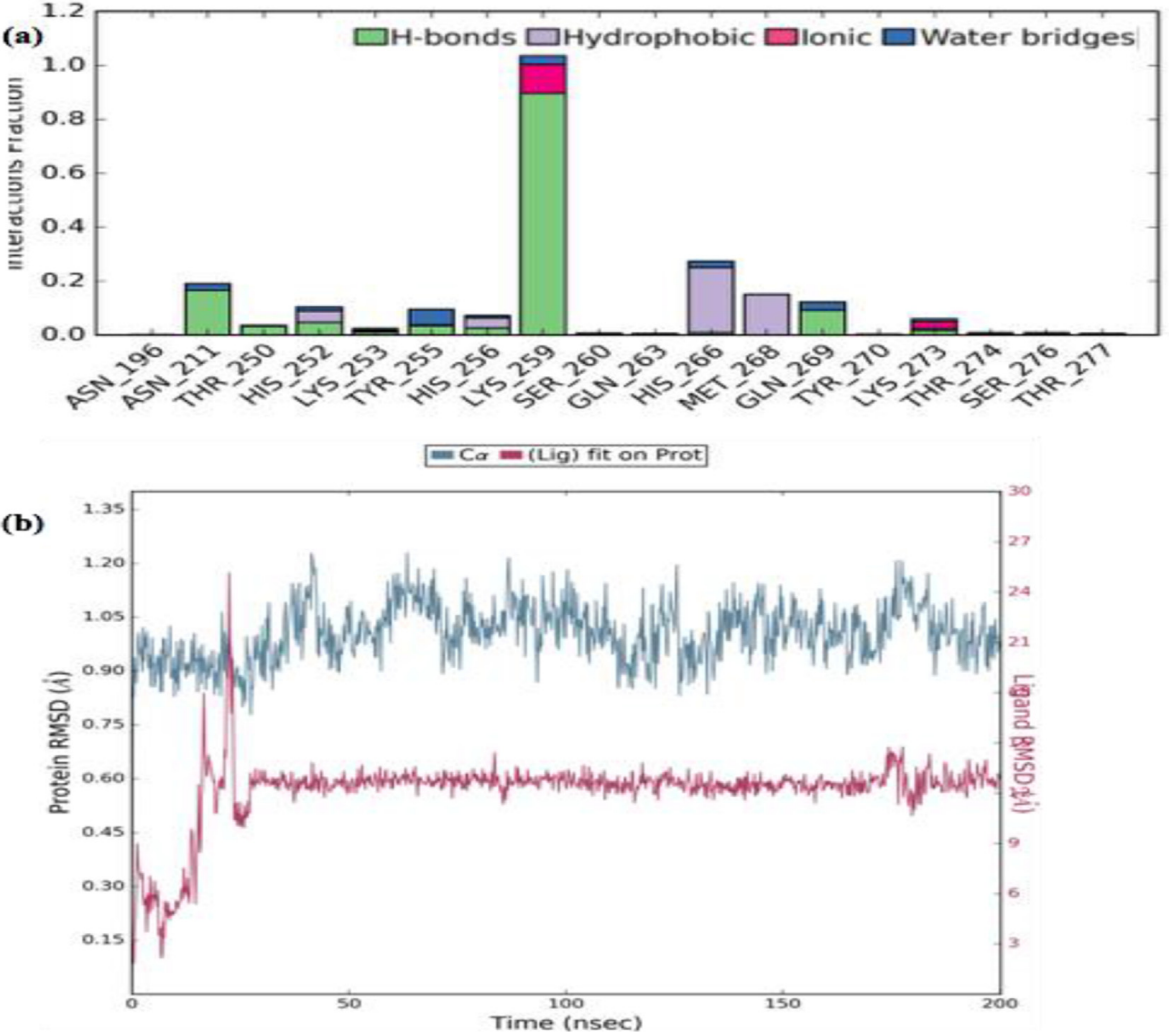
Interaction profile interims of δ-secretase-Oprea1 complex interaction lead have been monitored via Histogram (Hydrogen bond, hydrophobic and ionic) from simulation trajectory and **(b)**: RMSD plot of the backbone of the δ-secretase-Oprea1.

Figure 6a shows the Histogram plot and timeline of protein-ligand contact of the δ-secretase-Oprea1 complex. Histogram reveals the residues; viz., Asn 196, Asn 211, Thr 250, His 252, Lys 253, Tyr 255, His 256, Lys 259, Gln 269 interacted via hydrogen bonds while the residues viz., Lys 259 and Lys 273 also contributed via ionic bonds and salt bridges. Residues such as Tyr 255, His 266, and Gln 269 contributed via water bridges too, followed by hydrophobic interaction by His 266, Met 268, His 256, His 252 residues playing a pivotal role in deciphering the binding of Oprea1 into the active site pocket of δ-secretase. During the course of simulations, residues such as Asn 196, Asn 211, Thr 250, His 252, Lys 253, Tyr 255, His 256, Lys 259, Gln 269 were key interacting residues with Oprea1. Lys 259 is one of the key residues which maintained the hydrogen bond with Oprea1 during the entire course of 200ns simulations.

**Figure 7:**
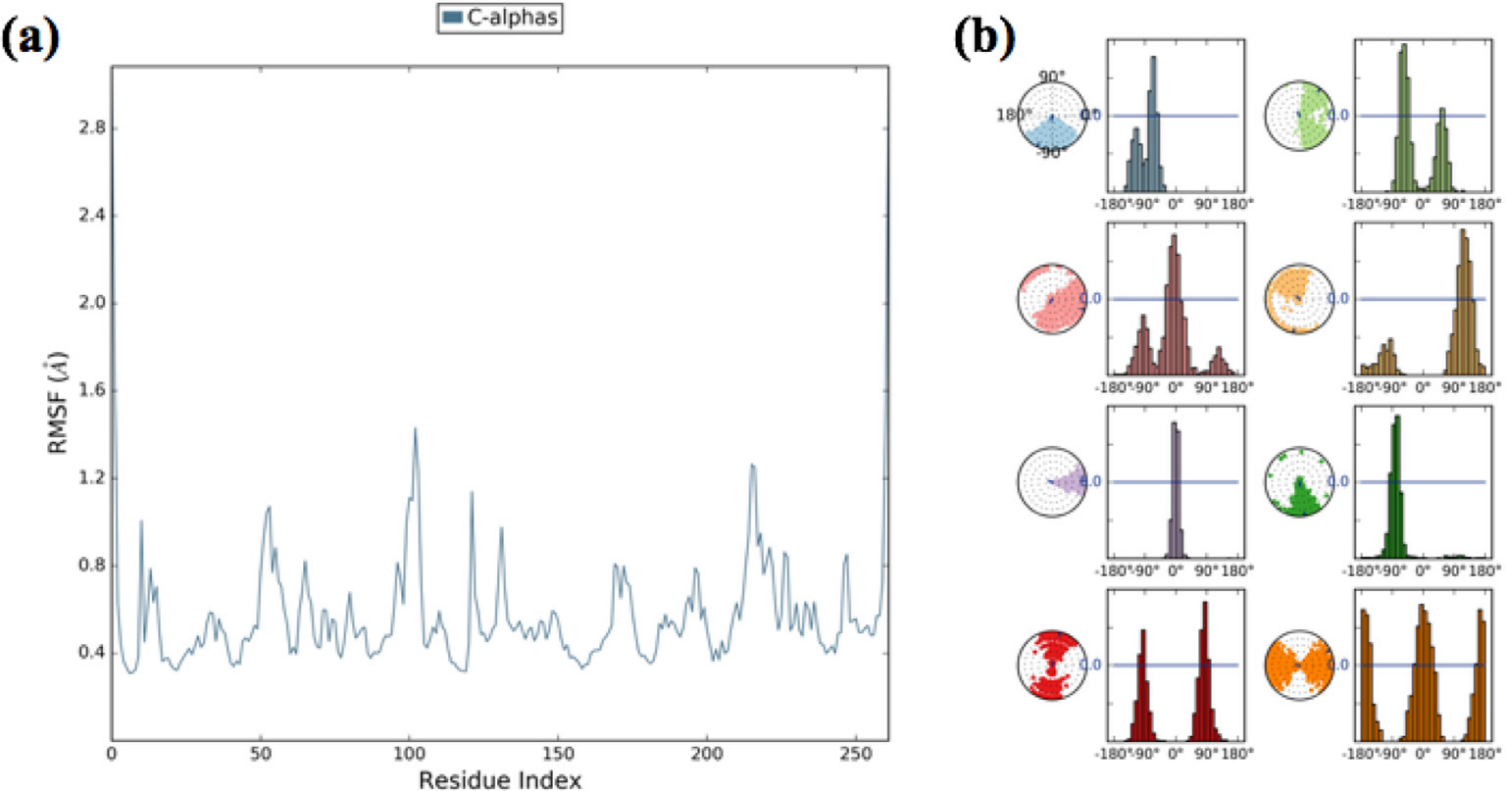
(a): RMSF of interaction profile in terms of δ-secretase-Oprea1 complex, while as (b): represents the probability density of torsion of δ-secretase-Oprea1 complex illustrated in terms of dial plots/radial plots, data so obtained has been plotted on bar plots (Histograms).

Structural deviation of the δ-secretase-Oprea1 complex was analyzed using RMSD of overall protein and RGYR during the course of 200ns. As shown in Figure 6b, RMSD of the protein Cα-backbone with respect to its starting position increased to 1.20 Å for the first 25 ns of the trajectory and then became stable around 0.6 Å in the last 170 ns course of the simulation. The radius of gyration (RGYR) as shown in Figure 5 reveals that compactness was maintained till the end of simulations. Further, RMSF as depicted in Figure 7a, depicts that the fluctuation found among the residues might be due to the presence of the loop. Thus, the conformational stability of the docked complex was analyzed which reveals that all complexes were highly stable, and provides the base for the interaction stability analysis being essential for our study to decipher the inhibitory potential of the Oprea1.

To illustrate the conformational changes of every ratable of δ-secretase-Oprea1 complex, a torsional profile was calculated during the simulation run of 0.00–200.00 ns. Figure 7b represents the probability density of torsion of Oprea1, illustrated in terms of dial plots, data so obtained has been plotted on bar plots (Histograms). Over a simulation period of 200 ns, the conformational torsions have been illustrated in terms of Radial or dial plots, wherein the initial simulation process is in the center of the dial plot and the evolution of time is plotted radials outward. Throughout the simulation time, Oprea1 was bound at the active site. RMSD of the Oprea1 complex remained consistent over the total simulation time.

**Figure 8:**
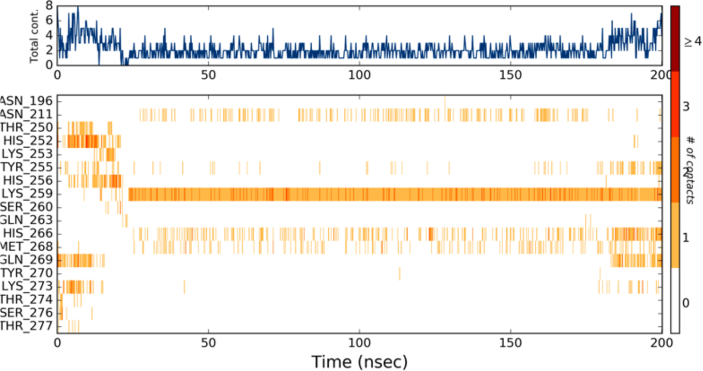
Protein-ligand contacts and ligand properties in the δ-secretase-Oprea1 complex.

Further, the protein-ligand contacts and ligand properties in the δ-secretase-Oprea1 complex were analyzed to determine the conformational as well the interaction stability of the docked complex and can be depicted in Figure 8. From the interaction profile, as shown in Figure 5, it is revealed that key residues like Asn 211, His 266, Met 268, Gln 269 maintain the binding with the δ-secretase; however, the interaction as provided by the Lys 259 were predominant and consistent throughout the simulation time of 200ns revealing its role in the binding of Oprea1 into the binding pocket of the δ-secretase.

### 3.7 Normal mode analysis & eigen values

**Figure S2:**
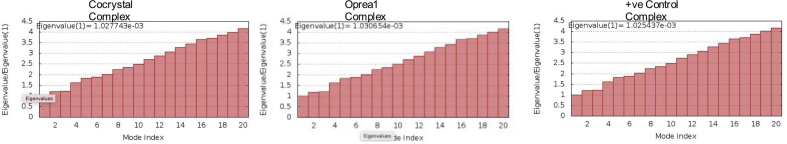
Eigen value of complexes.

**Figure S3:**
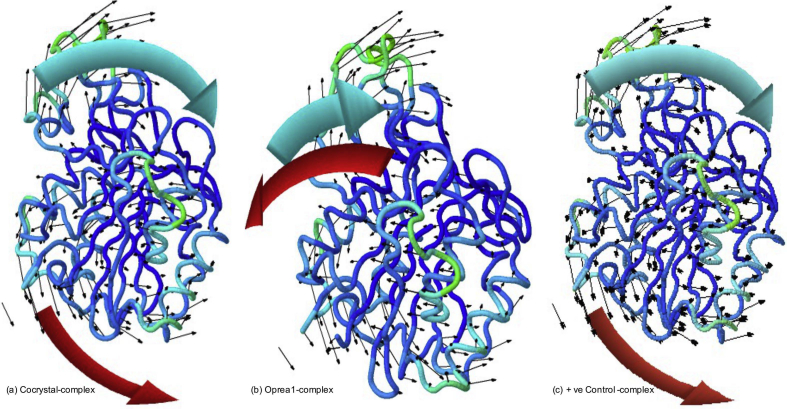
Domain motions of complexes.

Eigen values associated to each normal mode represent the motion softness, its value is directly related to the energy required to deform the structure. Normal mode analysis in terms of eigen value was investigated for cocrystal, Oprea1 and +ve control (Aloxistatin) complexes. The lower the eigen value the easier the deformation. Stability of the Oprea1 docked complex can be adumbarated by its eigen value. Data depicted in Supplementary section (Figure S2), reveals the stability of Oprea1 as that of +ve control (Aloxistatin) and cocrystal. The domain motions encoded by a single normal mode have been summarised with handful of curved arrows (Arrow field and Affine arrows), (Figure S3), where the longest path is depicted as wide curved arrow representing trajectories followed by each dynamical domain. The arrows and dynamical domains have are colored accordingly to improve the visualization of motions encoded in the mode.

## 4 Conclusion

Network analysis is effective for identifying features of AD in humans, as well as identifying genes that control pathological changes in network properties. We analyzed transcriptomic profiles using integrative multi-omics analysis to decipher system-level molecular signatures at the protein (hub proteins, TFs) and RNA level (miRNAs). We identified that LGMN and its associated genes are involved in multiple diseases. Network analysis of LGMN associated miRNAs lead to the identification of two novel miRNAs; hsa-miRNA-106a-5p and hsa-miRNA-34a-5p being highly expressed in the brain. Identification and expression of 10 Transcription factors associated with LGMN have also been unraveled. Spatio-temporal expression heatmap results show high and continuous expression of LGMN in most of the regions of the brain, especially in the frontal cortex. In a first-of-its-kind study, the LGMN gene-related regulatory miRNAs resulted in the identification of AD-specific TFs and miRNAs involved in AD. Our findings reveal the dysregulated miRNA: hsa-mir-26b-5p could be used as a biomarker in the diagnosis of AD. In parallel, IFD studies reveal that the Oprea1 bound well at the active site target of the δ-secretase. Residues like Thr 250, Hie 252, Hie 256, Lys 259, Gln 269 as hydrogen-bonded, while residues such as Met 268, Thr 274, Ser 276, Thr 277 interacted hydrophobically. Furthermore, Molecular dynamic simulations investigation of the δ-secretase-Oprea 1 docked complex have been carried out for a time period of 200 ns, revealed that Oprea1 has better binding affinity than that of 4-morpholin-4-yl-2,1,3-benzoxadiazol-7-amine reflected by RMSD, Rgyr, and RMSF profiles. Conformational stability of the docked complex confirms the high stability of Oprea1 with δ-secretase and provides the base for the interaction stability analysis being essential for our study to decipher the inhibitory potential of Oprea1. We have identified and analyzed the interactions of the target gene in the form of a network, and other important genes responsible for the modular existence of the network. Thus, based on Network analysis, the findings of our study ameliorate that identified miRNAs and Transcription Factors involved in simultaneous multiple associated diseases and multiple roles of LGMN in regulating the different pathways be further investigated for their potential role and implication in combatting AD and AD-associated diseases. Further studies are needed to establish dysregulation of mir-26b-5p in blood and brain, as biomarker for AD to have clinical utility.

## Declarations

### Author contribution statement

Saleem Iqbal, Md. Zubbair Malik: Conceived and designed the experiments; Performed the experiments; Analyzed and interpreted the data; Wrote the paper.

Debnath Pal: Contributed reagents, materials, analysis tools or data.

### Funding statement

This research did not receive any specific grant from funding agencies in the public, commercial, or not-for-profit sectors.

### Data availability statement

Data included in article/supplementary material/referenced in article.

### Declaration of interests statement

The authors declare no conflict of interest.

### Additional information

No additional information is available for this paper.

## Appendix A Supplementary data

Table S1 Gene enrichment. Table S1

Table S2 Dysregulation of miRNAs in Alzheimer's disease and targeted response. Table S2

Table S3 List of miRNA in the circulation and cerebral-spinal fluid (CSF) of AD patients. Table S3

Table S4 Differentially expressed miRNAs prefrontal cortex of brain. Table S4

Table S5 miRNA enrichment. Table S5
